# Ferroptotic alveolar epithelial type II cells drive T_H_2 and T_H_17 mixed asthma triggered by birch pollen allergen Bet v 1

**DOI:** 10.1038/s41420-024-01861-3

**Published:** 2024-02-23

**Authors:** Linyi Ma, Ying He, Huancheng Xie, Jing Wang, Jiaqian Chen, Shijie Song, Le Zhang, Linmei Li, He Lai, Yongping Liu, Huifang Chen, Xueyan Zhang, Xueting Liu, Zehong Zou, Qingling Zhang, Jie Yan, Ailin Tao

**Affiliations:** 1 The Second Affiliated Hospital, Guangdong Provincial Key Laboratory of Allergy & Immunology, The State Key Laboratory of Respiratory Disease, Guangzhou Medical University, 250 Changgang Road East, Guangzhou, 510260 China; 2 Department of Clinical Laboratory, General Hospital of the Yangtze River Shipping, Wuhan, 430005 China; 3 Guangdong Provincial Key Laboratory of Allergy & Immunology, Guangzhou Institute of Respiratory Health, National Clinical Research Center for Respiratory Disease, National Center for Respiratory Medicine, State Key Laboratory of Respiratory Diseases, The First Affiliated Hospital of Guangzhou Medical University, Guangzhou, 510120 China

**Keywords:** Cell death and immune response, Cell death

## Abstract

Asthma is a common allergic disease characterized by airway hypersensitivity and airway remodeling. Ferroptosis is a regulated death marked by iron accumulation and lipid peroxidation. Several environmental pollutants and allergens have been shown to cause ferroptosis in epithelial cells, but the relationship between birch pollinosis and ferroptosis in asthma is poorly defined. Here, for the first time, we have identified ferroptosis of type II alveolar epithelial cells in mice with Bet v 1-induced asthma. Further analysis revealed that treatment with ferrostatin-1 reduced T_H_2/T_H_17-related inflammation and alleviated epithelial damage in mice with Bet v 1-induced asthma. In addition, ACSL4-knocked-down A549 cells are more resistant to Bet v 1-induced ferroptosis. Analysis of clinical samples verified higher serum MDA and 4-HNE concentrations compared to healthy individuals. We demonstrate that birch pollen allergen Bet v 1 induces ferroptosis underlaid T_H_2 and T_H_17 hybrid asthma. Lipid peroxidation levels can be considered as a biomarker of asthma severity, and treatment with a specific ferroptosis inhibitor could be a novel therapeutic strategy.

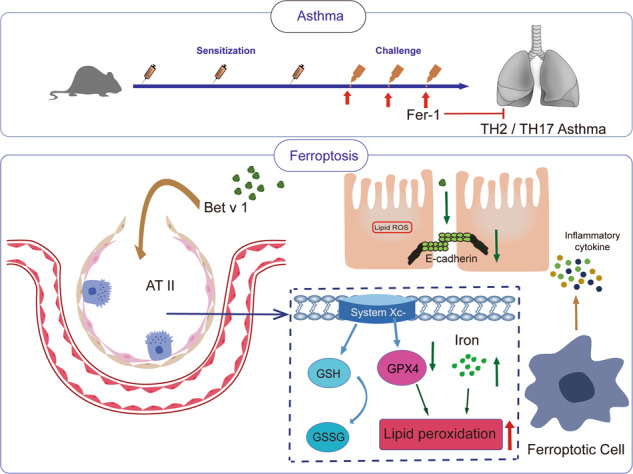

## Introduction

Asthma is a chronic inflammatory disease characterized by airway hyperreactivity (AHR) and airway remodeling that affects ~4.5% of adults worldwide [1, 2]. Pollen allergy affects >10% of the global population, and up to one-third of the affected individuals displaying hay fever symptoms will later develop allergic asthma [3]. Birch pollen, rupturing and releasing an aerosol ranging in size from 30 nm to 4 μm and containing Bet v 1 allergen, is a prominent elicitor of allergic sensitization and asthma [4, 5]. Oxidative stress, specifically lipid peroxidation, is thought to contribute to the pathophysiology of asthma [6]. Bet v 1 increases reactive oxygen species (ROS) levels and enhances inflammation independently of pollen-derived intrinsic NADPH oxidase activity, suggesting that additional factors are involved in the pollen-derived oxidative stress [7]. Cell death signaling has been reported to be modulated by oxidative stress [8]. There is thus adequate potential and value to explore the Bet v 1-induced cell death.

The term ferroptosis was coined in 2012 to describe a novel form of programmed cell death driven by lipid peroxidation [9]. The iron overload, ROS production as well as polyunsaturated fatty acids (PUFAs) supply are associated with iron-dependent lipid ROS accumulation, which induces ferroptosis [10]. The reactants of lipid peroxidation are PUFAs. Acyl-CoA synthetase long-chain family member 4 (ACSL4) is an essential isozyme for PUFAs production and dictates ferroptosis sensitivity [11, 12]. Glutathione peroxidase 4 (GPX4), a central regulator of ferroptosis, can oxidize two molecules of GSH into GSSG and reduce toxic lipid alcohol hydroperoxides [13]. The synthesis of GSH is mainly regulated by solute carrier family 7 member 11 (SLC7A11), the membrane transporter importing cysteine for glutathione biosynthesis [14]. It has recently been known that cystine/glutamate antiporter (xCT) promotes GSH and GPX4 protein synthesis [15]. Malondialdehyde (MDA) and 4-Hydroxynonenal (4-HNE) are reliable markers of lipid oxidation in clinical situations [16]. Previous studies have found that plasma MDA levels are a potential and predictive biomarker of asthma severity, suggesting the need to explore the relationship between ferroptosis and asthma [17, 18].

Epithelial cells, the essential physical barrier between the body and the environment, are prone to damage and even death when exposed to pollutants or allergens, which subsequently promote downstream immune responses [19, 20]. Epithelial integrity is related to E-cadherin expression, E-cadherin is a type I cadherin transmembrane glycoprotein coded by the *CDH1* gene and forms the main structure of apical junctional complexes and the epithelial barrier [21]. Sputum-soluble E-cadherin levels reflect the severity of asthma and are inversely correlated with decreases in FEV1 [22]. In cancer cells, E-cadherin-mediated intercellular interactions inhibit ferroptosis, suggesting a potential link between impaired epithelial barrier and ferroptosis.

The alveolar epithelium, which represents 99% of the surface area of the lungs, has significant defensive and immunoregulatory functions [23, 24]. Type II alveolar epithelial (AT II) cells, the progenitor cells for the alveolar epithelium, are responsible for remodeling the epithelial barrier and producing pulmonary surfactant [25]. AT II cells can produce and secrete a variety of chemokines and cytokines in response to infection or damage [26]. Meanwhile, it has been established that AT II cells express MHC II and inhibit T cells from future activation in an Ag-dependent manner [27]. Therefore, the study of AT II cells may aid the understanding of pulmonary inflammatory diseases, especially in allergic asthma.

Here, our findings reveal that Bet v 1 inhalation-induced ferroptosis in the AT II cells is associated with ACSL4. Treatment with a specific ferroptosis inhibitor effectively ameliorated Bet v 1-induced asthma in mice. We first validate ferroptosis in type II alveolar epithelial cells in asthma, and lay the groundwork for future therapeutic strategies targeting ferroptosis.

## Results

### Higher levels of lipid peroxidation are prevalent in patients with asthma

**Fig. 1:**
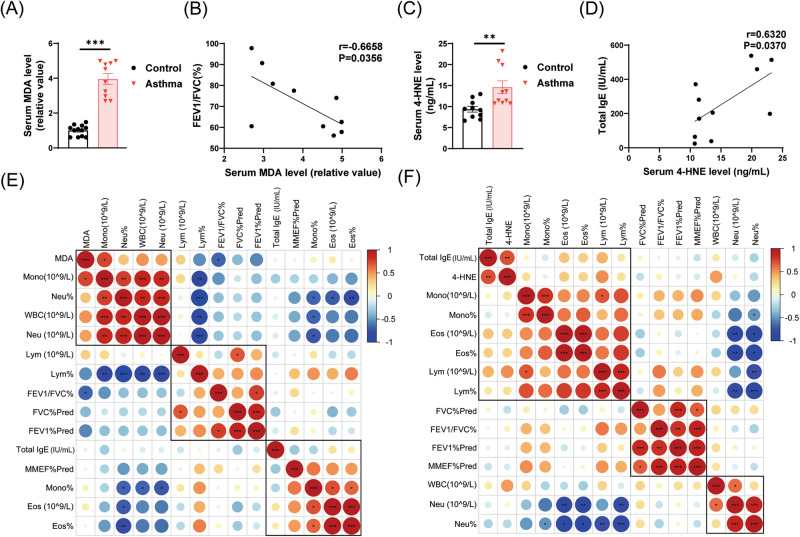
Higher levels of lipid peroxidation are prevalent in patients with asthma. **A**, **B**, **E** MDA level is normalized to volume and expressed by a relative ratio with the mean of the control group. **A** Serum MDA level in asthma patients (*n* = 10) and healthy controls (*n* = 12). **B** Linear correlation between serum MDA and the FEV1/FVC ratio. **C** Serum 4-HNE level in asthma patients (*n* = 10) and healthy controls (*n* = 10). **D** Linear correlation between serum 4-HNE and the serum total IgE (IU/mL). **E**, **F** Correlation analysis between serum MDA or 4-HNE and routine blood test as well as pulmonary function. Data are presented as mean ± SEM. *P < 0.05; **P < 0.01; ***P < 0.001.

To substantiate the notion that higher levels of lipid peroxidation are prevalent in asthma patients, we examined the major lipid peroxidation product levels in asthma patients. A total of 10 patients with asthma were included in the MDA analysis (Table S1), and another 10 patients with asthma were included in the 4-HNE analysis (Table S2). Compared with healthy controls, the levels of serum MDA and 4-HNE in asthma patients were significantly increased (Fig. 1A, C). We analyzed the correlation of various indicators in patients with asthma (Fig. 1E, F). It was noteworthy that the serum MDA levels correlated inversely with the ratio of the forced expiratory volume in the first second to the forced vital capacity of the lungs (FEV1/FVC) (Fig. 1B), and the serum 4-HNE levels were positively associated with serum total IgE levels (Fig. 1D). Considering that lower FEV1/FVC and higher IgE levels are positively correlated with asthma severity, we suggest that lipid peroxidation levels may be used as an indicator of asthma severity.

### Excess lipid peroxidation and elevated iron levels in Bet v 1-induced asthmatic mice

**Fig. 2:**
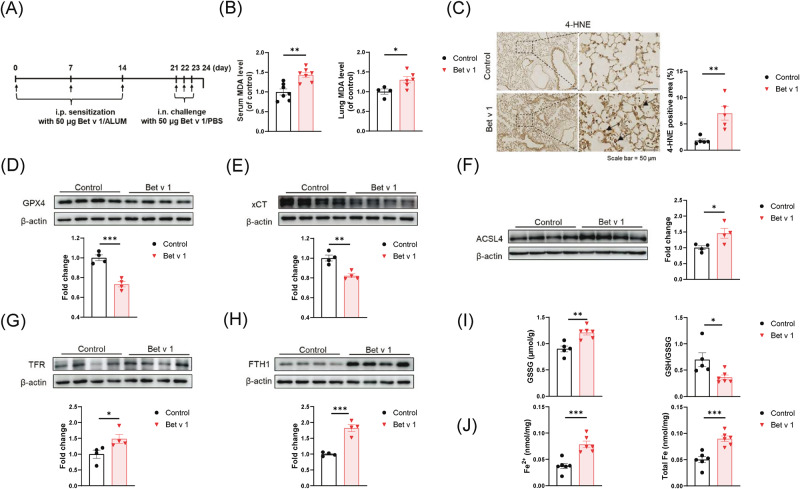
Increased lipid peroxidation and iron levels in Bet v 1-induced asthmatic mice. **A** Schematic diagram of the construction of Bet v 1-induced asthmatic mice model. **B** Serum and Lung MDA levels in mice. The lung MDA level was normalized to tissue quality, and the serum MDA level was normalized to volume. **C**–**J** Samples from the lungs of Bet v 1-induced mice and control group. **C** The expression of 4-HNE determined by immunohistochemical staining. **D**–**H** The proteins levels of GPX4, xCT, ACSL4, TFR, and FTH1. The protein expression level was normalized to β-actin. **I** The levels of GSSG and GSH/GSSG in mice lung tissue measured by GSSG/GSH Quantification Kit. **J** The concentration of free iron and total iron in mice lung tissue measured by Iron Assay Kit. Data are presented as mean ± SEM. *P < 0.05; **P < 0.01; ***P < 0.001.

We further explored this phenomenon in mice. To more closely match human asthma disease, we chose Bet v 1 recombinant protein (Fig. S1A), the major allergen of birch pollen, to build a mouse asthma model (Fig. 2A). AHR, HE, PAS staining, and cytokine levels in bronchoalveolar lavage fluid (BALF) were consistent with the pathological characteristics of mixed-type asthma of T_H_2 and T_H_17 (Fig. S1B–E). Bet v 1 induced higher MDA levels both in serum and lung (Fig. 1B). The immunohistochemical staining of 4-Hydroxynonenal (4-HNE), a stable marker for lipid peroxidation, was stronger than the control group (Fig. 1C). Subsequently, we detected the levels of key proteins in mice lungs (Fig. 2D–H). Lower anti-lipid peroxidation proteins GPX4 and xCT reflected loss of antioxidant capacity (Fig. 2D, E). Higher ACSL4 levels increased susceptibility to ferroptosis (Fig. 2F). Given that increased free iron plays a critical role in the Fenton reaction during ferroptosis, we assessed whether Bet v 1 increases the amount of labile iron available for ferroptosis. The western blot and Immunohistochemistry analysis revealed that Bet v 1 stimulated the expression of transferrin receptor (TFR) and ferritin heavy chain 1 (FTH1) in lung tissue, indirectly reflecting the increase in iron level (Fig. 2G, H). The iron assay results showed significantly higher contents of iron in Bet v 1-exposed lung homogenates, including ferric iron and total iron (Fig. 2J). Additionally, Bet v 1 exposure led to higher GSSG levels and lower GSH/GSSG ratio, which were markers of oxidative stress and toxicity (Fig. 2I). Overall, these results point to an excess of lipid peroxidation in mice with Bet v 1-triggered asthma.

### Ferroptosis is involved in the AT II cells of Bet v 1-induced asthmatic mice

**Fig. 3:**
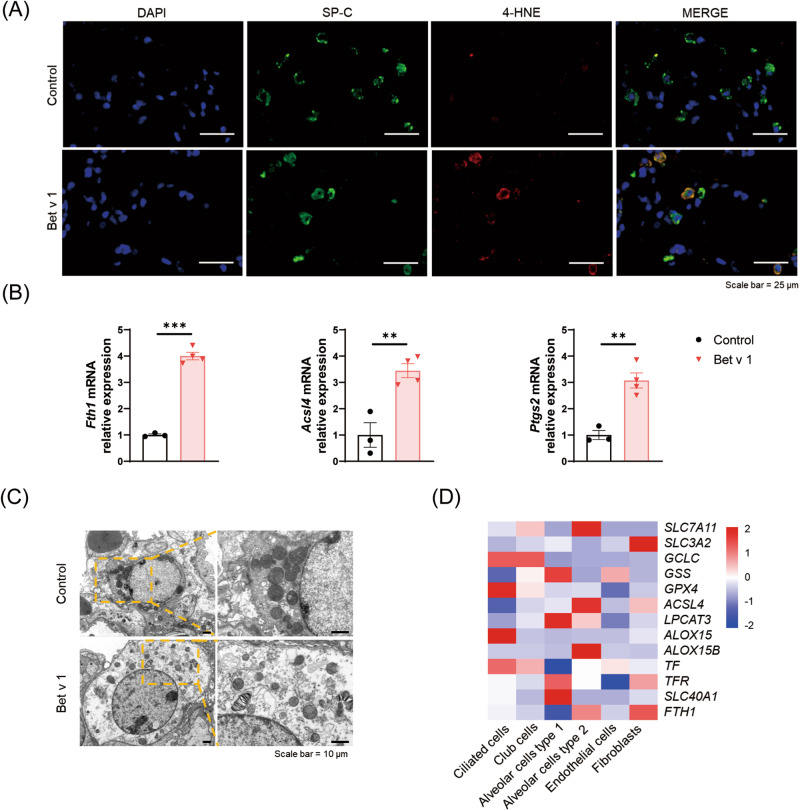
AT II cells showed elevated 4-HNE level and abnormal mitochondrial morphology. **A** Immunofluorescence staining of SP-C and 4-HNE in lungs from Bet v 1-induced asthmatic mice and control. **B** The mRNA levels of *Fth1*, *Acsl4*, *Ptgs2* in AT II cells isolated from mice lungs. **C** Representative images of mitochondrial injury in the lung of Bet v 1-induced asthmatic mice under indicated treatment were observed by TEM. **D** Heatmap of the ferroptosis-related genes mRNA levels in lung epithelial and mesenchymal cells in the HPA database. Data are presented as mean ± SEM. *P < 0.05; **P < 0.01; ***P < 0.001.

Immunohistochemical staining of 4-HNE showed higher signaling in lung interstitium compared with the control group (Fig. 2C). Meanwhile, immunofluorescence staining showed that 4-HNE was mainly detected in SP-C^+^ cells, rather than EMBP^+^ or LY6G^+^ cells, indicating aberrant lipid peroxidation in AT II cells (Figs. 3A, S2A, B). AT II cells were isolated from mice by a procedure modified by Corti et al. [28]. There were up-regulated mRNA levels of *Fth1*, *Acsl4* as well as *Ptgs2* in AT II cells from asthmatic mice (Fig. 3B). The type II alveolar epithelial cells (AT II cells) are large cuboidal cells located at the corners of alveoli. To further confirm the ferroptosis in AT II cells, we monitored the morphological changes using transmission electron microscopy. In Bet v 1-induced asthma mice, AT II cells exhibited shrinkage of mitochondria and increase in membrane density, features unique to ferroptosis (Fig. 3C). Based on the Human Protein Atlas (HPA) database, lower level of GPX4 and higher level of ACSL4 and ALOX15B (Fig. 3D) were detected in AT II cells than other epithelia, indicating that AT II cells exhibit enhanced susceptibility to ferroptosis. All these results demonstrated that type II alveolar epithelial cells suffered from ferroptosis in Bet v 1-induced asthma.

### Bet v 1 induces ferroptosis associated with ACSL4 expression in AT II cell lines

**Fig. 4:**
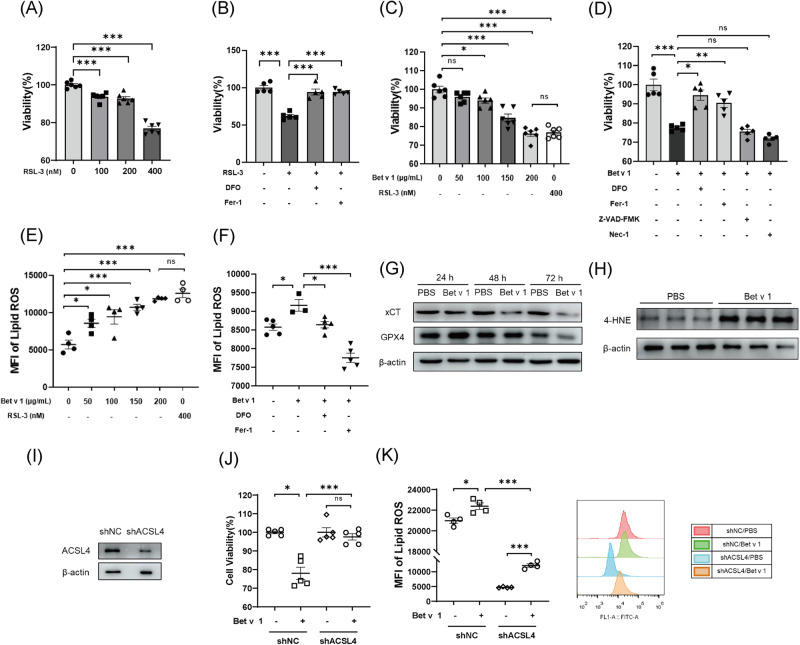
Bet v 1 induces ferroptosis associated with ACSL4 expression in AT II cell lines. **A**–**D** A549 cells were treated as indicated for 30 hours, and the cell viability was detected by CCK-8 assay. **E**, **F** The A549 cells were treated as indicated for 30 hours (**E**) or 24 hours (**F**), and lipid ROS levels of different groups were determined by the C11 BODIPY lipid peroxidation sensor. **G** The protein levels of xCT and GPX4 in A549 cells treated with 200 μg/mL Bet v 1 or PBS for 24, 48, or 72 hours. **H** The protein levels of 4-HNE in A549 cells treated with 200 μg/mL Bet v 1 or PBS for 24 hours. **I** The ACSL4 expression level in A549 cell transfected with shNC or shACSL4. **J**, **K** After being treated with 200 μg/mL Bet v 1 or PBS, cell viability and lipid ROS were assessed. Data are presented as mean ± SEM. ns, not significant; *P < 0.05; **P < 0.01; ***P < 0.001.

We stimulated A549 cells, human type II alveolar epithelial cell lines, with a concentration gradient of RSL-3 or Bet v 1 for 30 hours, and measured cell survival rate by cell counting kit-8 (CCK-8). Cell mortality is related to protein or drug concentration (Fig. 4A, C). Cell survival rate increased when treated with ferroptosis inhibitors ferrostatin-1 (Fer-1) and Deferoxamine mesylate (DFO) rather than apoptosis or necroptosis inhibitors (Fig. 4B, D). The levels of lipid peroxidation in cells increased with the concentration gradient of Bet v 1, as measured by C11-BODIPY 581/591 staining (Fig. 4E). Ferrostatin-1 and DFO significantly inhibited lipid ROS accumulated induced by Bet v 1 (Fig. 4F). Western blot analysis showed that Bet v 1 up-regulated level of 4-HNE and down-regulated xCT and GPX4 expression in A549 cells (Fig. 4G, H). In vitro experiments demonstrated that Bet v 1 reduced the expression of GPX4 and induced ferroptosis in AT II cell lines.

ACSL4 was highly expressed in AT II cells both in humans (Fig. 3D) and mice (Fig. S2C). We hypothesize that ACSL4 sensitizes AT II cells to Bet v 1-induced ferroptosis. After knocked down ACSL4 (Fig. 4I), A549 cells were resistant to Bet v 1-induced ferroptosis and had lower levels of lipid ROS (Fig. 4J, K).

### Ferrostatin-1 therapy reduces lipid peroxidation and attenuates Bet v 1-induced asthma

**Fig. 5:**
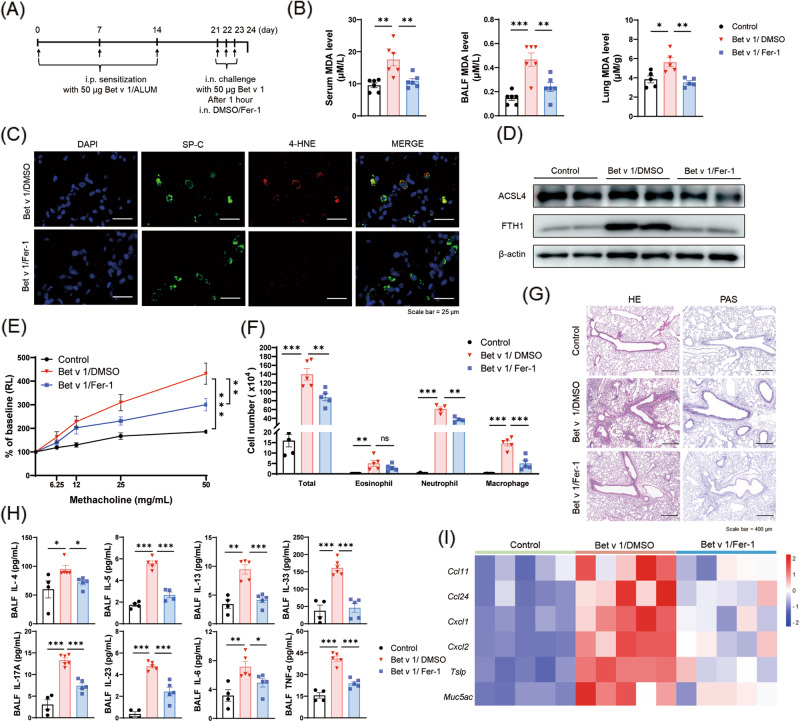
Ferrostatin-1 treatment reduced lipid peroxidation and attenuated Bet v 1-induced asthma. **A** Fer- 1 (20 mg/kg) or DMSO was administered intranasally 1 hour before each challenge. **B** MDA levels of serum, BALF, and lung in mice. **C** Immunofluorescence staining of SP-C and 4-HNE in lungs. **D** The protein levels of ACSL4 and FTH1 in mice lungs. **E** Airway hyper reactivity assessment in the indicated groups. **F** Amounts of the total cell, eosinophil, neutrophil, and macrophage in BALF. **G** HE and PAS staining in lung sections. **H** Cytokine levels in BALF by ELISA. **I** The mRNA levels in lung tissues of *Ccl11, Ccl24, Cxcl1, Cxcl2, Tslp, and Muc5ac* in the indicated groups. Data are presented as mean ± SEM. ns, not significant; *P < 0.05; **P < 0.01; ***P < 0.001.

Considering the potential clinical application, we tested the therapeutic effect of Ferrostatin-1 (Fer-1) on Bet v 1-induced asthmatic mice (Fig. 5A). Treatment of Fer-1 reduced MDA levels of serum, BALF, and lung (Fig. 5B). Immunofluorescence staining assay showed that Fer-1 reduced 4-HNE level in AT II cells (Fig. 5C). Western blot assay showed that lower ACSL4 and FTH1 levels after treatment with Fer-1 (Fig. 5D). Fer-1 treatment strikingly attenuated AHR and alleviated the pathological injury including peribronchial and perivascular inflammatory cell infiltration as well as mucus secretion (Fig. 5E, G). Meanwhile, significantly decreased amounts of total cells, eosinophil, neutrophil and macrophage in BALF were observed in the mice treated with Fer-1 (Fig. 5F). Moreover, ELISA assays revealed that the concentrations of T_H_2 and T_H_17-related cytokines including IL-4, IL-5, IL-13, IL-33, IL-17A, IL-23, IL-6 and TNF-α in BALF were down-regulated after the treatment of Fer-1 (Fig. 5H). Furthermore, intratracheal administration of Fer-1 decreased the mRNA expression levels of *Ccl11, Ccl24, Cxcl1, Cxcl2,* and *Tslp* and further reduced the infiltration of inflammatory cells in the lung (Fig. 5I). MUC5AC, gel-forming mucin and a member of the secreted glycoprotein family, is thought to contribute to the airway hyperresponsiveness in asthma patients [29, 30]. The intervention of Fer-1 reduced the mRNA level of *Muc5ac*, which was consistent with the results of AHR and PAS staining (Fig. 5I). The above data suggest that Fer-1 reduces airway inflammation and ameliorates Bet v 1-induced asthma.

### The treatment with Ferrostatin-1 restored E-cadherin levels and alleviated epithelial damage

**Fig. 6:**
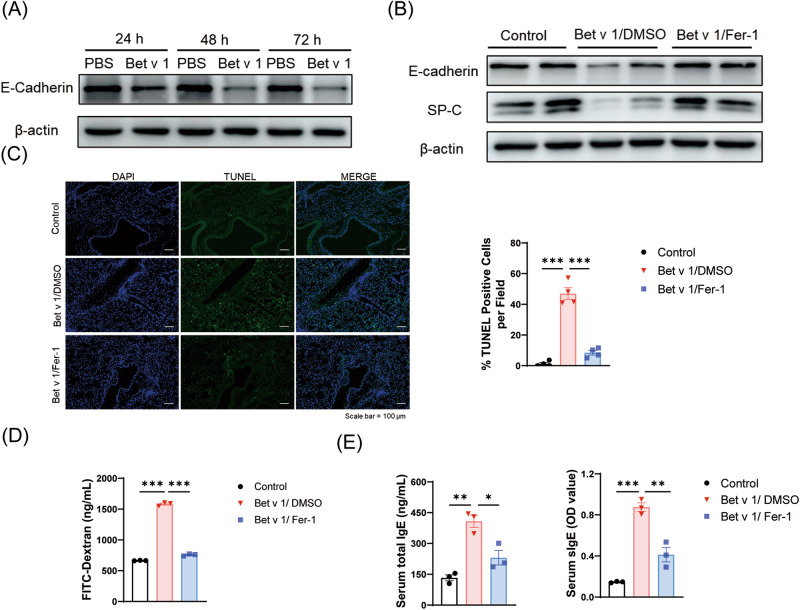
Ferrostatin-1 treatment restored E-cadherin levels and alleviated epithelial damage. **A** The protein levels of E-cadherin in A549 cells treated with 200 μg/mL Bet v 1 or PBS for 24, 48, or 72 hours. **B** The protein levels of SP-C and E-cadherin in the lung of Bet v 1-induced asthmatic mice. **C** TUNEL fluorescence staining and statistical analysis of cell death in mice lung sections. **D** Lung permeability is measured by the amount of FITC-Dextran through the airway into the serum. **E** The total IgE and Bet v 1 specific IgE in sera. Data are presented as mean ± SEM. *P < 0.05; **P < 0.01; ***P < 0.001.

Damage to the epithelial barrier gives rise to increased exposure of allergens to immune cells. Epithelial cadherin (E-cadherin) is a transmembrane protein that provides important structural and immune functions to the airway epithelium. E-cadherin levels in A549 cells co-incubated with Bet v 1 were significantly down-regulated (Fig. 6A). SP-C is involved in activating the immune response of alveolar macrophages to infection and plays a critical role in the maintenance and innate defense of the lungs [31]. The restoration of E-cadherin and SP-C illustrated that Fer-1 effectively alleviated epithelial damage and restored epithelial functions (Fig. 6B). The TUNEL assay showed an increase in cell death when exposed to Bet v 1. Fer-1 medication remarkably suppressed the TUNEL-positive cell rate in airway and pulmonary interstitial (Fig. 6C). In addition, we assessed epithelial permeability using FITC-dextran nasal drops. The concentration of FITC-dextran in serum was lower after Fer-1 treatment, indicating that Fer-1 mitigated epithelial barrier damage (Fig. 6D). Levels of IgE were significantly elevated in allergic asthma, reflecting higher body sensitivity. The serum total IgE and Bet v 1-specific IgE levels were significantly lower in the Fer-1 treated mice (Fig. 6E). These results suggest that Fer-1 may reduce allergic reactions and ameliorate asthma.

## Discussion

Asthma is a heterogeneous disorder that is causally classified into Type-2 asthma and non-Type-2 asthma based on pathological and immunological features [32, 33]. Upon initial allergen exposure, oxidative stress may be a critical mechanism for eliciting allergic sensitization. Ferroptosis is also a type of death caused by oxidative stress. The limitations of related studies are being reflected in the deepening understanding of ferroptosis in asthma. Most studies only hook on airway epithelial cells/cell lines, ferroptosis in other cells is readily overlooked. For the first time, we focus on the ferroptosis in a new cell type, alveolar type II cells, which play a pivotal role in asthma. Alveolar epithelial cells have important defensive and immunoregulatory functions. Although more attention is paid to airway epithelial cells when asthma is mentioned, damage to alveolar epithelial cells is a driving factor in asthma [19, 24]. Once damaged, alveolar epithelial cells are able to secrete the cytokines IL-6, TNF-α, and various chemokines such as CXCL1 and CXCL2 to alter the inflammatory cells [34, 35]. Different from other airway epithelial cells, AT II cells express high levels of ACSL4. We find that ACSL4-knocked-down A549 cells are resistant to Bet v 1-induced ferroptosis and have lower levels of lipid ROS, suggesting that ACSL4 sensitizes AT II cells to ferroptosis, which also corroborates previous studies [12]. The level of ACSL4 was up-regulated in our Bet v 1-induced asthma mice model, which is consistent with other reports [36]. It is an intriguing question for future research to investigate whether ACSL4-dictated ferroptosis in AT II cells is prevalent in asthma induced by different allergens.

The therapeutic effect of ferroptosis inhibitors on asthma has been demonstrated in HDM and OVA induced asthma mouse model [37, 38]. Our study complements research on the role of ferroptosis inhibitors in pollen-involved asthma. More than 100 million people worldwide suffer from birch pollen allergies, most of whom are also affected by secondary pollen food syndrome [4, 39]. Bet v 1, the major potent allergen of birch pollen, is a main cause of allergic rhinitis and asthma symptoms, which belongs to pathogenesis-related class 10 (PR-10) proteins, characterized by a hydrophobic cavity that can bind to plentiful small molecule ligands [40]. Bet v 1, therefore, is homologous and cross-reactive to diverse allergens from different families and genuses, such as *Betulaceae* (alder, hornbeam, hophornbeam, hazel), *Fagales* (beech, chestnut, oak), *Rosaceae* (apple, almond, peach), *Apiaceae* (celery, fennel, carrot), and even *Fabaceae* (soybean, peanut), etc. [41–45]. This cross-reactivity is based primarily on the sequence homology and extensive IgE binding capacity of allergen homologs to the major birch allergen Bet v 1 [41, 46, 47]. Due to Bet v 1-allergic individuals exhibiting IgE cross-reactivity to allergens from a diverse range of plant species, a more comprehensive treatment regimen is required. Hopefully, individual allergen Bet v 1 immunotherapy can reduce systemic allergic immune responses and airway inflammation of modeled mice with allergic asthma [48], while rBet v 1B2, a hypoallergenic folding variant of Bet v 1, presented substantially lower numbers of eosinophils in the BALF of the mice, showing better effects on immunotherapy [49]. In the future, the use of non-allergenic or hypoallergenic proteins as a control could explore whether the ability to induce ferroptosis is associated with allergen sensitization. Predictably, ferroptosis inhibitors and even ferroptosis-related recombinant allergen-specific immunotherapies may yield novel therapeutic options for asthma patients.

Altered iron levels have been linked to respiratory diseases [50]. Our study also confirmed a significant increase in the amount of iron contents in the lungs of mice with Bet v 1-induced asthma. Bet v 1 is capable of iron binding via catechol-based siderophores, and it is worth exploring whether this is associated with the higher iron levels in Bet v 1-exposed mice [51].

Remarkably, ferroptosis stress has been attributed to elevated levels of ROS and impaired antioxidant defenses in patients with asthma [52]. It is, therefore, tempting to suggest that ferroptosis in alveolar type II cells would be one of the starting points for secondary inflammation in asthma. In the current study, the inhibition results justify this proposal. Fer-1 attenuates the infiltration of inflammatory cells and the airway hyperreactivity induced by Bet v 1. Apparently, the AHR and airway inflammation in Bet v 1-induced asthmatic mice were not completely abolished by Fer-1 of the ferroptosis inhibitor (Fig. 5). Quercetin, a kind of natural dietary flavonoid that ameliorates neutrophilic airway inflammation by alleviating ferroptosis and M1 macrophage polarization, also fails to completely extinguish the airway inflammation induced by LPS [53]. All these data suggest that the non-ferroptosis factors also play a role in the agitation of inflammation in the airways of asthmatic subjects.

Our study also has important implications for the specific objects of ferroptosis. In fact, it is difficult to generalize the effect of ferroptosis intervention strategies on asthma control. Protective effects of ferroptosis inhibitors have also been reported in HDM and OVA-induced asthma by mitigating oxidative damage [38]. However, the ferroptosis-inducing agents (FINs), such as erastin, Ras-selective lethal small molecule 3, artesunate, etc., can induce non-canonical ferroptosis of eosinophils in vitro and in vivo, able to relieve asthma inflammation. Due to their distinct mechanism from Dexamethasone (DXMS)-induced apoptosis, FINs exhibit synergistic effects with DXMS in asthma treatment [54]. The inhibitory Fer-1 and inductive FINs of ferroptosis play the same role in asthma control by affecting different cells. It would therefore be intriguing to decipher the effects of the sequential use of both agents for asthma control. It is important to note that the direct effect of FINs on the structure and function of DXMS is indeterminate. Treatment with ferroptosis inducers or inhibitors requires a more complete understanding of the properties of the drug itself. Targeted therapy is the key point of future research. Future investigations using AT II cell-targeted ferroptosis inhibitor complexes have the potential to shed light on novel functions of AT II cells and to avoid the effects of ferroptosis on other cells in practical applications [55].

Intact epithelial barriers are essential for the maintenance of tissue homeostasis, as they protect host tissues from infections, pollutants, and allergens. Bet v 1 reduces the expression of E-cadherin in vivo and in vitro. In epithelial cells, E-cadherin mediates intercellular interactions and suppresses ferroptosis by activating the intracellular NF2 and Hippo pathways. Antagonizing this axis can promote ferroptosis by upregulating several ferroptosis modulators, including ACSL4 and TFRC [56]. Whether Bet v 1-induced lower levels of E-cadherin influence the initiation or exacerbation of ferroptosis deserves further investigation in the future. We were able to confirm, at least, that Fer-1 reduces epithelial damage during asthma development and thus ameliorates asthma. However, asthma often has repeated attacks as a chronic disease. Whether Fer-1 can relieve asthma after the disease develops needs further investigation.

Fer-1 reduces the levels of cytokines and chemotactic factors. The efficacy of Fer-1 in mice with Bet v 1-induced asthma further suggests that the lipid peroxidation stress may amplify the inflammatory response. The loss of function of the adhesion G-protein coupled receptor ADGRF5 or the endoplasmic reticulum chaperone GRP78 induces mucous cell metaplasia, fibrosis, and Type-2 immune response [57, 58]. Type-2 inflammation is likely to promote the loss of function of ADGRF5 and GRP78 in a positive feedback manner, thus creating a vicious circle of chronic airway inflammation and making asthma refractory.

Collectively, ACSL4-dependent ferroptosis in alveolar epithelial type II cells facilitates Bet v 1-induced T_H_2/T_H_17-related inflammatory and epithelial damage, orchestrating allergic disease and being capable of acting as an intervention target for allergy therapy.

## Materials and methods

### Mice and treatment

The six- to eight-week-old female wild-type C57BL/6 mice were purchased from Beijing SiPeiFu Biotechnology Limited Company. All mice were maintained on a 12-hour light/dark cycle under specific pathogen-free conditions, with free access to sterile water and irradiated food. All studies were conducted in accordance with the guidelines of the committee of Guangzhou Medical University on the use and care of animals and were authorized by the Animal Subjects Committee of Guangzhou Medical University (Approval No. A2019-042).

On days 0, 7, and 14, C57BL/6 mice were sensitized intraperitoneally with 200 μL PBS for the control group or 200 μL emulsion of Bet v 1 and ALUM for the experimental group. On days 21, 22, and 23, the mice were challenged intranasally with PBS or Bet v 1 (Fig. 2A). Fer- 1 (20 mg/kg), DMSO, or PBS was administered intranasally 1 hour before each challenge (Fig. 5A).

### Patient samples

The sera of patients with asthma were selected from a serum bank set up in the Allergy Department of the Second Affiliated Hospital of Guangzhou Medical University. Health checkups were conducted at the Second Affiliated Hospital of Guangzhou Medical University, and people with healthy checkup data were enrolled as healthy controls. The study was approved by the Ethics Committee of the Second Affiliated Hospital of Guangzhou Medical University. Informed consent was obtained from all enrolled subjects.

### Recombinant Bet v 1

Plasmid vector pET-44 EK/LIC including Bet v 1 sequence (UniProtKB P43179), previously constructed in our laboratory, was transformed into *ClearColi*® BL21 (DE3) Electrocompetent Cells (#60810-1, Lucigen, USA), the competent cells with a modified LPS that does not trigger the endotoxic response. *ClearColi*® BL21 Cells were incubated with shaking at 37 °C overnight in LB medium containing 100 μg/mL ampicillin, and the protein expression was induced by 1 mM isopropyl-β-d-thiogalactoside (IPTG). Harvested cells were resuspended in binding buffer (100 mM Tris-HCl, 150 mM NaCl, 1 mM EDTA, pH 8.0) and lysed with mild sonication. Protein refolding and purification were performed as described previously [59]. Recombinant proteins were stored at -20 °C. The concentration of the protein was determined by BCA Protein Assay Kit (#23225, Thermo Fisher, USA). The purity of Bet v 1 was verified by staining with Coomassie brilliant blue.

### Airway hyperresponsiveness measurements

24 hours after the last challenge, airway reactivity to methacholine was assessed by the FinePointe Resistance and Compliance system (DSI-Buxco, St. Paul, MN, USA). The response to inhaled methacholine (0, 6.25, 12.5, 25, and 50 mg/mL) was evaluated by measuring lung resistance (RL) for 5 minutes after each nebulization step. Results are expressed as a percentage of baseline RL value (value at 0 mg/mL methacholine).

### Immunohistochemistry and Immunofluorescence

Tissues were collected and fixed in 4% paraformaldehyde for 24 hours and embedded in paraffin. Lung sections (4 μm) were stained with hematoxylin and eosin (HE) staining and periodic acid-Schiff (PAS) staining to evaluate the pathological damage. The expressions of EMBP (sc-365701, Santa Cruz, USA), LY6G (#ab25377, Abcam, USA), 4HNE (#ab211326, Abcam, USA), and SP-C (#ab211326, Abcam, USA) were characterized by immunohistochemistry or immunofluorescence using specific antibodies.

### Western blot

Total proteins from lung tissues and cells were extracted using RIPA lysis buffer (Cat #P0013C, Beyotime, China). Proteins were separated using SDS-PAGE, and transferred to polyvinylidene fluoride (PVDF) membranes (#1620177, Bio-Rad, USA). The membranes were incubated with specific primary antibodies against 4-HNE (#ab46545, Abcam, USA), TFR (P02786, Abmart, China), FTH1 (sc-376594, Santa Cruz, USA), SP-C (#ab211326, Abcam, USA), GPX4 (#ab125066, Abcam, USA), ACSL4 (sc-365230, Santa Cruz, USA) and β-actin (Cat # RM2001, Beijing Ray Antibody Biotech, China) overnight at 4 °C. The membranes were washed and incubated with goat anti-mouse IgG-HRP (Cat # RM3001, Beijing Ray Antibody Biotech, China) or goat anti-rabbit IgG-HRP (Cat # RM3002, Beijing Ray Antibody Biotech, China) for 1 hour at room temperature. The protein bands were exposed to an Amersham Imager 680 UV for image capture.

### Real-time quantitative PCR analysis

Total RNA was extracted by Trizol reagent (Cat #: T9108, Takara, Japan) and reverse-transcribed to complementary DNA with Thermo Scientific RevertAid Master Mix (#M1632, Thermo Fisher, USA). The PCR reaction was carried out on Light Cycler 480 Fast Real-Time PCR System using PowerUp™ SYBR™ Green Master Mix (#A25742, Applied Biosystems, USA). The primer sequences used are shown in Table S3.

### Flow cytometry

For classification of granulocytes, cells from BALF were stained with Zombie Aqua™ solution for 10 min in the dark, stained for 30 min with antibodies for markers as follows: FITC Rat Anti-Mouse CD45 (#553079, BD, USA), PE/Cyanine7 anti-mouse CD11c (#117317, BioLegend, USA), BB700 Rat Anti-Mouse CD11b (#566416, BD, USA), APC Rat Anti-Mouse Ly-6G (#560599, BD, USA), PE Rat Anti-Mouse Siglec-F (#552126, BD, USA), BV421 Rat Anti-Mouse F4/80 (#565411, BD, USA). Absolute cell counts were calculated on the basis of Precision Count Beads^TM^ (#424902, BioLegend, USA). Data were collected on a BD Biosciences FACSVerse Flow Cytometer and analyzed using FlowJo software. The Flow cytometry gating strategy is shown in Supplementary Fig. S1F.

### Cytokine analysis

Levels of cytokines in the BALF supernatants were measured using ELISA kits following the manufacturer’s instructions. The ELISA kits used were bought from Thermo Fisher, USA, their detailed information is as follows: IL-4 Mouse Uncoated ELISA Kit (#88-7013-88), IL-5 Mouse Uncoated ELISA Kit (#88-7054-88), IL-13 Mouse Uncoated ELISA Kit (#88-7439-88), IL-17A (homodimer) Mouse Uncoated ELISA Kit (#88-7371-88 A), IL-23 Mouse Uncoated ELISA Kit (#88-7230-88), IL-6 Mouse Uncoated ELISA Kit (#88-7066-88), IL-33 Mouse Uncoated ELISA Kit (#88-7333-88) and TNF alpha Mouse Uncoated ELISA Kit (#88-7324-88).

### Serum IgE detection

To test total IgE levels, mouse sera samples were diluted 10-fold and measured using IgE Mouse Uncoated ELISA Kit (#88-50460-88, Thermo Fisher, USA) according to the manufacturer’s instructions. Serum Bet v 1-specific IgE assays were detected as follows. Briefly, plates were coated with Bet v 1 and incubated with mouse sera for 2 hours at room temperature, followed by incubation with HRP-conjugated goat anti-mouse IgE antibodies (#PA184764, Thermo Fisher, USA, 1:1000). The reaction was stopped with 2 M H_2_SO_4_ followed by visualization with the added substrate TMB. The absorbance was immediately measured at 450 nm.

### Cell culture

A549 cells were cultured at 37 °C under 5% CO2 in DMEM (Gibco, USA) supplemented with 10% fetal bovine serum (Gibco, USA).

### Plasmid construction and transfection

The plasmids were synthesized by GenePharma. A549 cells were transfected with shACSL4 or shNC and selected with puromycin (2 μg/mL).

The sequences are listed as shACSL4: 5’- GGATATGATGCACCTCTTTGC-3’; shNC: 5’-TTCTCCGAACGTGTCACGT-3’. The results of regulated ACSL4 expression were tested by immunoblotting.

### Cell viability assay

A549 cells were plated into a 96-well plate and grown overnight, and then incubated with Bet v 1 or RSL-3 (HY-100218A, MCE, USA) at the indicated concentration in the presence or absence of 20 μM Fer-1 (HY-100579, MCE, USA), 20 μM DFO (HY-B0988, MCE, USA), 100 μM Z-VAD-FMK (HY-16658B, MCE, USA), 100 μM Nec-1 (T1847, Topscience, USA). Afterwards, cell counting kit-8 (CCK-8) solution (Cat #C0005, Topscience, China) was applied to each well, and the plate was subjected to one hour of incubation. The absorbance was determined at 450 nm.

### Lipid ROS detection

The cells were seeded in 48-well culture plates and treated as described above. Then cells were washed twice and incubated at 37 °C for 30 minutes with 1 μM C11-BODIPY 581/591 probe (D3861, Thermo Fisher, USA).

### MDA assay

Levels of MDA were determined with the MDA Assay Kit (BC0025, Solarbio, China) according to the manufacturer’s instructions.

### Iron assay

The levels of iron in lung tissue were measured by Iron Assay Kit (I291, Dojindo, Japan) according to the manufacturer’s instructions.

### Transmission electron microscopy

The fresh lung tissue was fixed with a solution containing 2.5% glutaraldehyde in 0.1 M phosphate buffer at 4 °C and photographed using a transmission electron microscope (TEM, HITACHI HT7700 120 kV).

### TUNEL assay

The death cells in mice lung sections were measured by TUNEL Apoptosis Detection Kit (YEASEN,40307ES60) according to the manufacturer’s instructions.

### Lung permeability assay

Mice were nasal inhaled with 10-kDa fluorescein isothiocyanate (FITC)-Dextran (GLPBIO). The fluorescence intensity of plasma was measured after 1 h (Ex = 495 nm; Em = 525 nm).

### Statistics analysis

Statistical analysis was performed using GraphPad Prism 8.0 (GraphPad Software) and presented as means ± SEM. Unpaired Student’s *t* tests, Mann–Whitney test, and one-way analysis of variance analysis were used in data analysis. The Pearson correlation coefficient was used for the correlation analysis of the two factors. R (version 4.2.0, R Foundation for Statistical Computing, Vienna, Austria) and RStudio (version Desktop 2022.07.2 + 576, PBC&B Corps, Boston, MA, USA) were used for clustering analysis. The cluster analysis was performed using Corrplot (Version 0.92, 2021, Taiyun Wei and Viliam Simko) for visualization. For animal experiments, mice of the same age and similar body weight were blinded and randomly divided into different groups. At least three mice per group to ensure adequate power, and all of the animal data were included for analysis. A similar variation of each group/treatments was estimated before statistical analysis. *P* < 0.05 is considered statistically significant.

### Reporting summary

Further information on research design is available in the Nature Research Reporting Summary linked to this article.

### Supplementary information

Supplementary Tables

Supplementary Figure

Original Data File

Reporting Summary

## Data Availability

All datasets generated and analyzed during the course of this study are included in this published article and its Supplementary Information files. Additional data are available from the corresponding author upon reasonable request.
