# Supplementary material for: Ferroptotic alveolar epithelial type II cells drive TH2 and TH17 mixed asthma triggered by birch pollen allergen Bet v 1
Source: Cell Death Discov. 2024 Feb 23;10:96. doi: 10.1038/s41420-024-01861-3 (PMC10891108; doi:10.1038/s41420-024-01861-3)
Supplement: Supplementary file 1 — Supplementary Tables [file 41420_2024_1861_MOESM1_ESM.docx]

**Supplementary Tables**

**Tables S1 Routine blood test and pulmonary function analysis of the patients enrolled**

|  | **Item** | **Median (P25, P75)** | **Normal range** |
| --- | --- | --- | --- |
| **Routine blood test** | WBC (10^9^ /L) | 6.55 (6.15, 7.88) | 4 - 10 |
|  | Neutrophil (10^9^ /L) | 3.50 (2,95, 4.55) | 1.8 - 8 |
|  | Lymphocyte (10^9^ /L) | 2.10 (2.00, 2.28) | 1.1 - 3.2 |
|  | Monocyte (10^9^ /L) | 0.55 (0.43, 0.68) | 0.1 - 0.6 |
|  | Eosinophil (10^9^ /L) | 0.30 (0.15, 0.48) | 0.05 - 0.3 |
|  | Neu% | 53.70 (46.48, 58.80) | 40 - 70 |
|  | Lym% | 31.65 (29.03, 33.23) | 20 - 40 |
|  | Mono% | 8.00 (7.60, 8.58) | 3 - 10 |
|  | Eos% | 5.60 (2.03, 9.28) | 0 - 7 |
|  | Total IgE | 302.50 (147.5, 619.75) | 0 - 10 |
| **Pulmonary function analysis** | FVC%Pred | 87.65 (70.58, 93.70) | ≥92 |
|  | FEV1%Pred | 65.90 (42.15, 83.25) | ≥80 |
|  | FEV1/FVC (%) | 68.34 (60.51, 79.98) | ≥80 |
|  | MMEF%Pred | 39.00 (15.70, 52.36) | ≥65 |

**Supplementary Tables**

**Tables S2 Routine blood test and pulmonary function analysis of the patients enrolled**

|  | **Item** | **Median (P25, P75)** | **Normal range** |
| --- | --- | --- | --- |
| **Routine blood test** | WBC (10^9^ /L) | 7.93 (6.95,8.28) | 4 - 10 |
|  | Neutrophil (10^9^ /L) | 4.35 (3.63, 5.88) | 1.8 - 8 |
|  | Lymphocyte (10^9^ /L) | 2.10 (1.70, 2.63) | 1.1 - 3.2 |
|  | Monocyte (10^9^ /L) | 0.50 (0.40, 0.58) | 0.1 - 0.6 |
|  | Eosinophil (10^9^ /L) | 0.25 (0.11, 0.44) | 0.05 - 0.3 |
|  | Neu% | 58.00 (45.63, 71.23) | 40 - 70 |
|  | Lym% | 31.10 (20.45, 31.98) | 20 - 40 |
|  | Mono% | 6.05 (5.10, 8.35) | 3 - 10 |
|  | Eos% | 2.95 (1.30, 5.50) | 0 - 7 |
|  | Total IgE | 243.00 (90.50, 437.00) | 0 - 10 |
| **Pulmonary function analysis** | FVC%Pred | 92.05 (69.68, 101.48) | ≥92 |
|  | FEV1%Pred | 67.95 (48.95, 80.63) | ≥80 |
|  | FEV1/FVC (%) | 75.99 (64.45, 88.83) | ≥80 |
|  | MMEF%Pred | 30.54(13.80,48.78) | ≥65 |

**Supplementary Tables**

**Tables S3 Primer Information**

| **Primer name (mouse)** | **Site** | **Sequence (5’—3’)** |
| --- | --- | --- |
| *acsl4* | F | TGAACGTATCCCTGGACTAGG |
|  | R | TCAGACAGTGTAAGGGGTGAA |
| *β-actin* | F | TGTTACCAACTGGGACGACA |
|  | R | GGGGTGTTGAAGGTCTCAAA |
| *ccl11* | F | TGCTCACGGTCACTTCCTTC |
|  | R | CTTGAAGACTATGGCTTTCAGGGTG |
| *ccl24* | F | ATTCTGTGACCATCCCCTCAT |
|  | R | TGTATGTGCCTCTGAACCCAC |
| *cxcl1* | F | GTGAGGACATGTGTGGGAGG |
|  | R | CACGTGCGTGTTGACCATAC |
| *cxcl2* | F | TCCAGAGCTTGAGTGTGACG |
|  | R | GCAAACTTTTTGACCGCCCT |
| *fth1* | F | TGAAGCTGCAGAACCAACGAGG |
|  | R | GCACACTCCATTGCATTCAGCC |
| *gpx4* | F | GCAACCAGTTTGGGAGGCAGGAG |
|  | R | CCTCCATGGGACCATAGCGCTTC |
| *muc5ac* | F | CAGGACTCTCTGAAATCGTACCA |
|  | R | AAGGCTCGTACCACAGGGA |
| *ptgs2* | F | TGCACTATGGTTACAAAAGCTGG |
|  | R | TCAGGAAGCTCCTTATTTCCCTT |
| *tslp* | F | CTGAGAGAAATGACGGTACTCAGG |
|  | R | CACCATAATGGAGCTACGTTAGAGC |

Note: F, forward; R, reverse.
