# Supplementary material for: Ferroptotic alveolar epithelial type II cells drive TH2 and TH17 mixed asthma triggered by birch pollen allergen Bet v 1
Source: Cell Death Discov. 2024 Feb 23;10:96. doi: 10.1038/s41420-024-01861-3 (PMC10891108; doi:10.1038/s41420-024-01861-3)
Supplement: Supplementary file 2 — Supplementary Figure [file 41420_2024_1861_MOESM2_ESM.pdf]

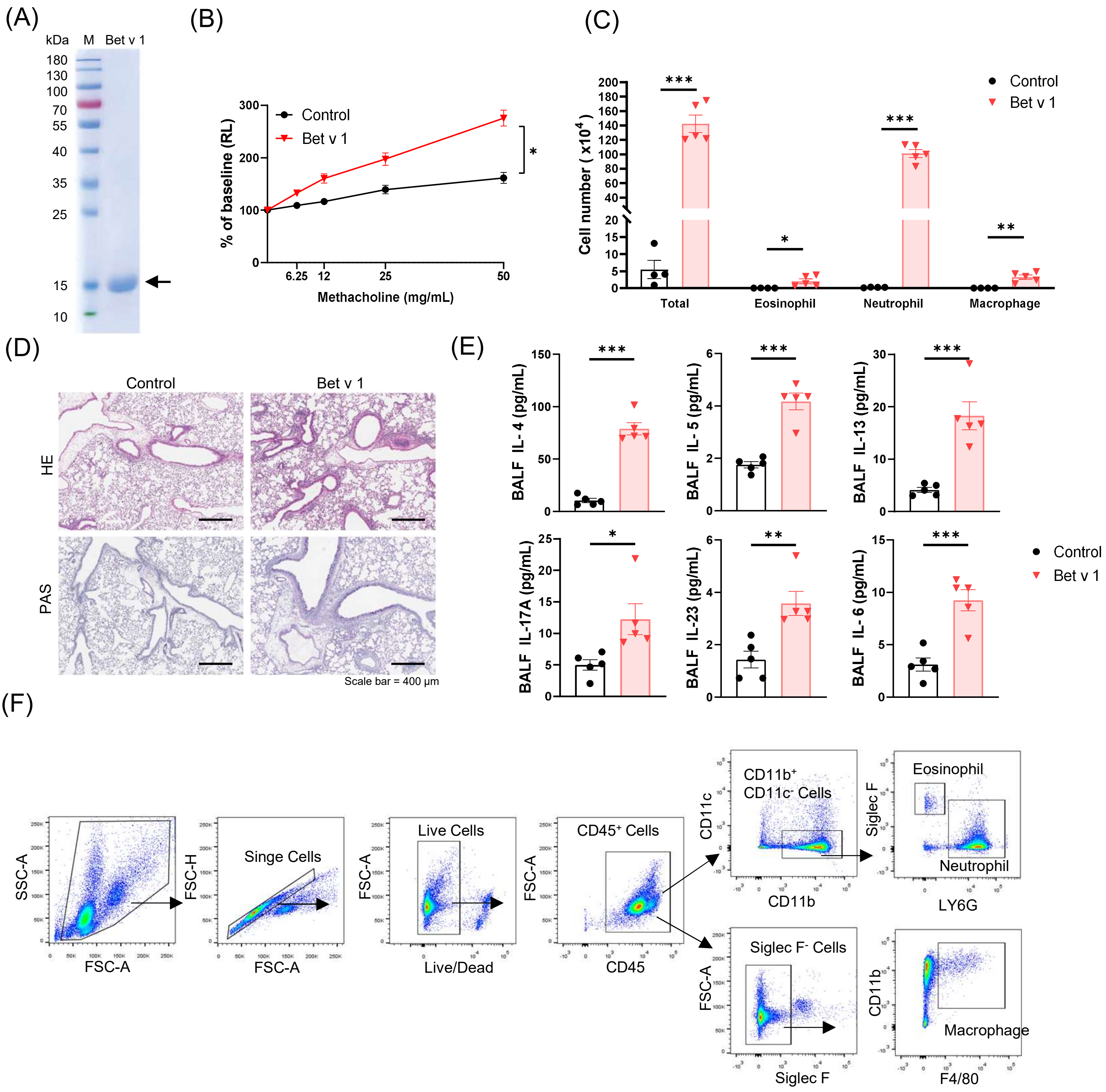

Figure S1

Figure S1. Bet v 1 induced asthmatic mice model. (A) The purity of Bet v 1 was determined by Coomassie brilliant blue. (B-E) Characterization of Bet v 1-induced asthmatic mouse model. (B) Airway hyperresponsiveness assessment in the indicated groups. (C) Amounts of the total cell, eosinophil, neutrophil and macrophage in BALF. (D) HE and PAS staining in lung sections. (E) Cytokine levels in BALF. (F) Flow cytometry gating strategy for eosinophil, neutrophil and macrophage in BALF. Data are presented as mean ± SEM. *P < 0.05. **P < 0.01; ***P < 0.001.

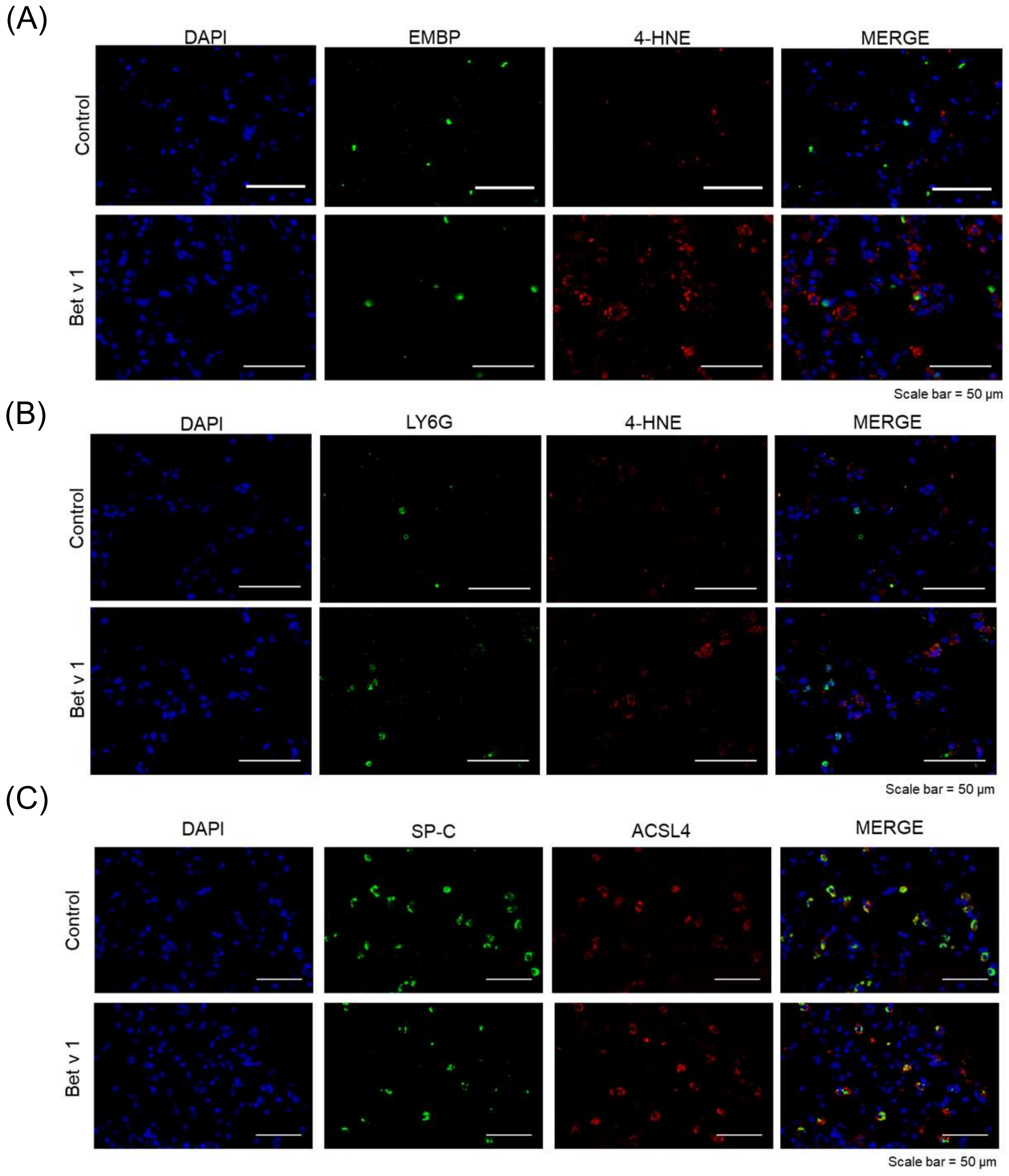

Figure S2

Figure S2. The expression level of 4-HNE in different cells. (A-B) The immunofluorescence staining of EMBP, LY6G and 4-HNE in lungs of Bet v 1-induced asthmatic mice and control. (C) Immunofluorescence staining of SP-C and ACSL4 in lungs Bet v 1-induced asthmatic mice and control.
