# Supplementary material for: Ferroptotic alveolar epithelial type II cells drive TH2 and TH17 mixed asthma triggered by birch pollen allergen Bet v 1
Source: Cell Death Discov. 2024 Feb 23;10:96. doi: 10.1038/s41420-024-01861-3 (PMC10891108; doi:10.1038/s41420-024-01861-3)

Fig2D β-actin

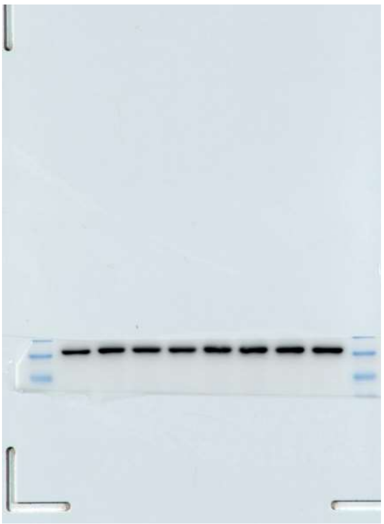

Fig2D GPX4

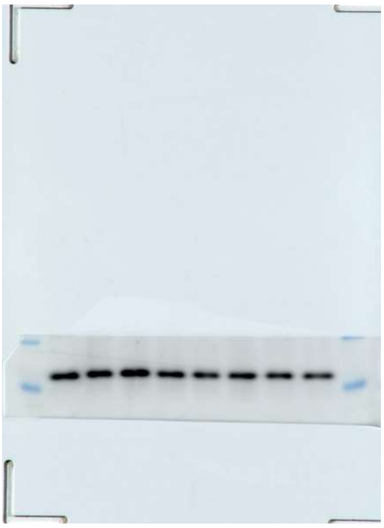

Fig2e β-actin

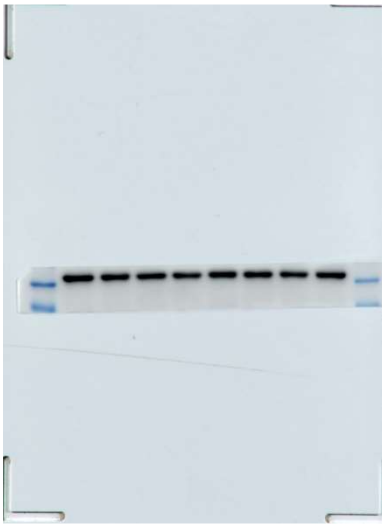

Fig2e xCT

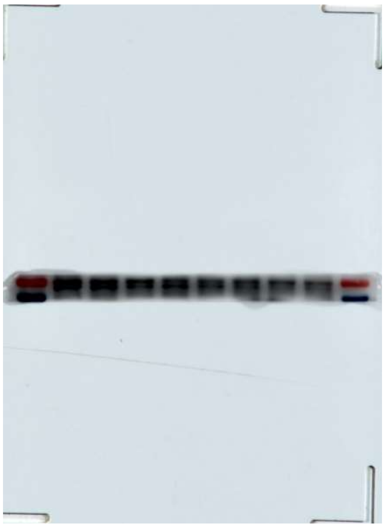

Fig2F β-actin

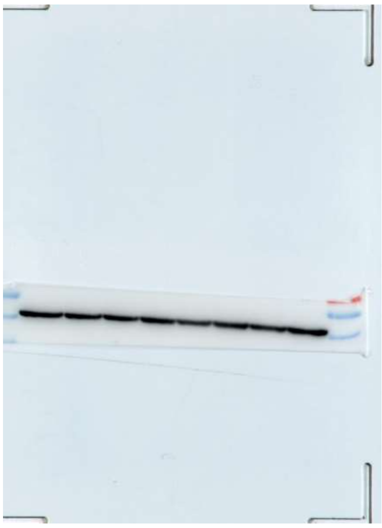

Fig2F ACSL4

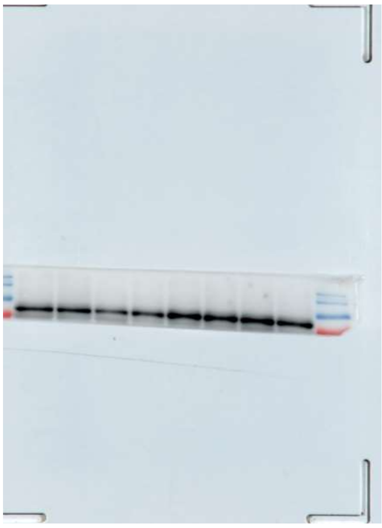

Fig2G β-actin

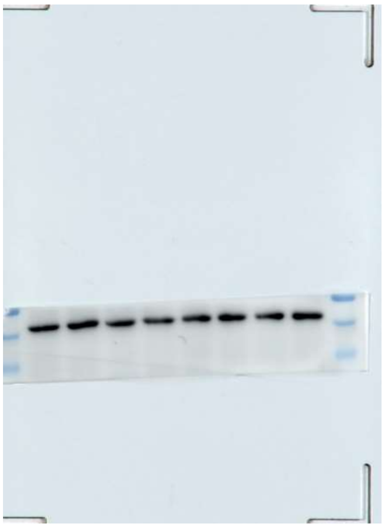

Fig2G TFR

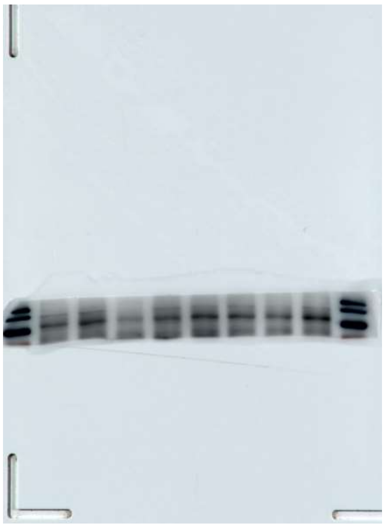

Fig2H β-actin

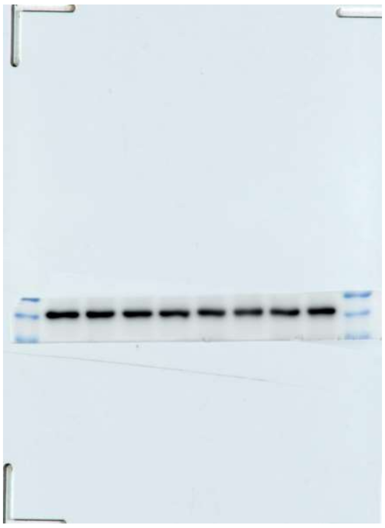

Fig2H FTH1

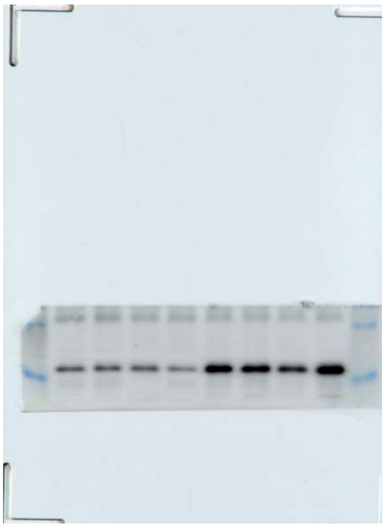

Fig4G β-actin

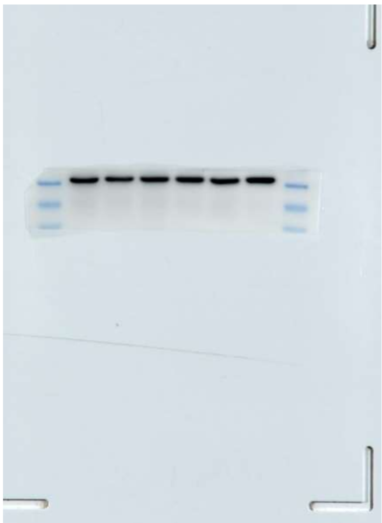

Fig4G xCT

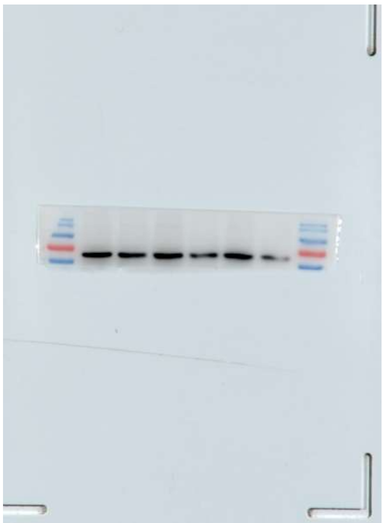

Fig4G GPX4

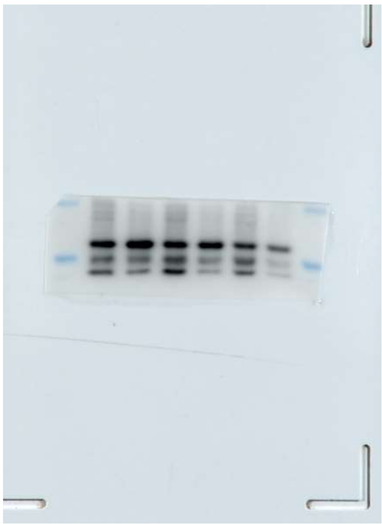

Fig4H β-actin

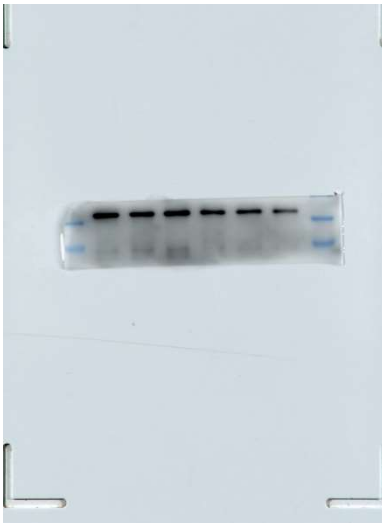

Fig4H 4-HNE

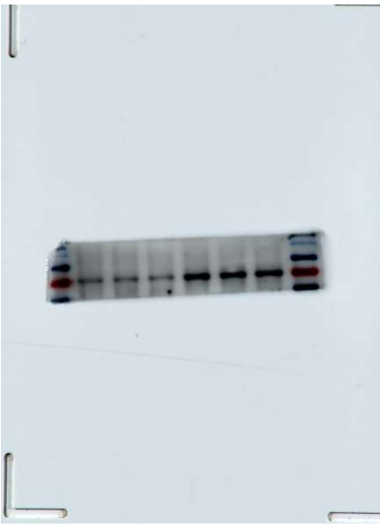

Fig4I β-actin

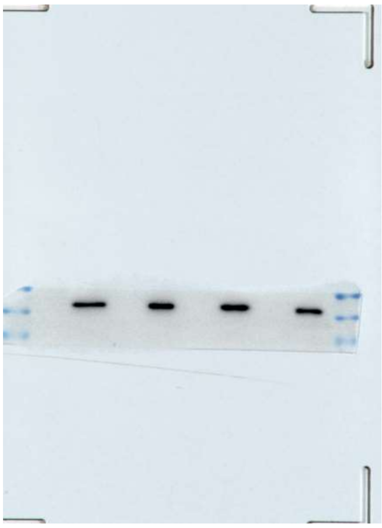

Fig4I ACSL4

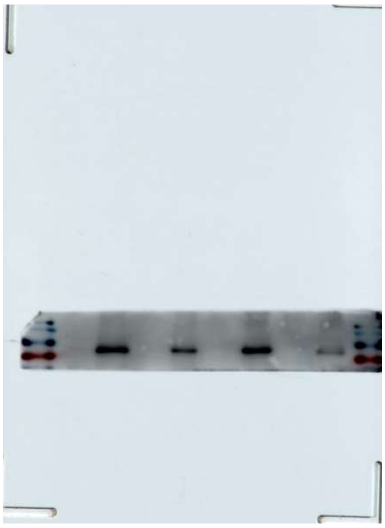

Fig5D β-actin

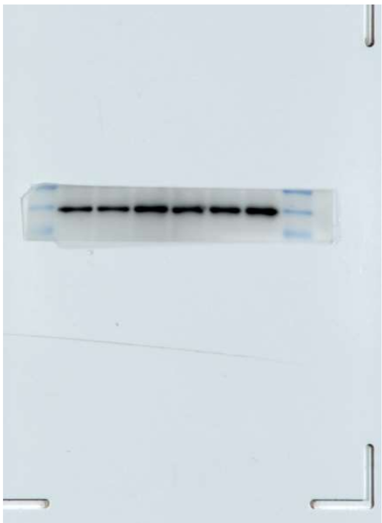

Fig5D ACSL4

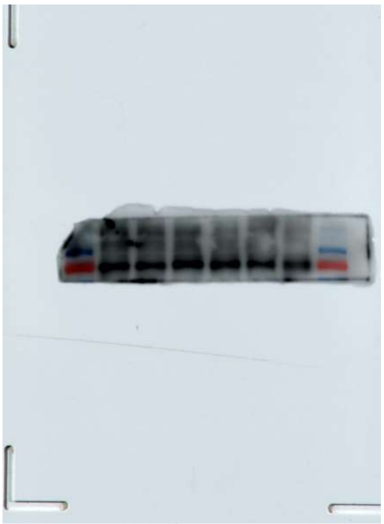

Fig5D FTH1

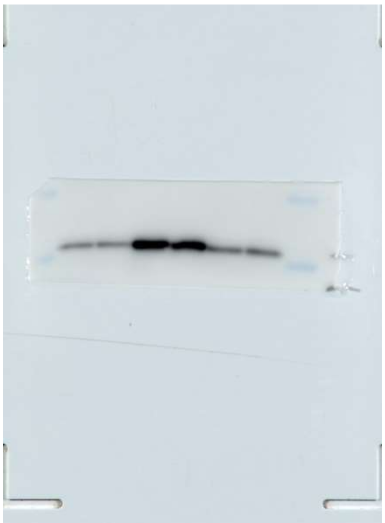

Fig6A β-actin

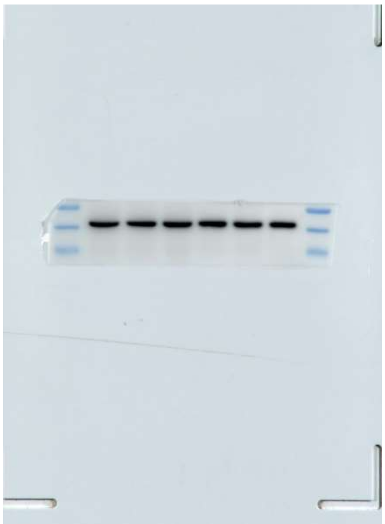

Fig6A E-cadherin

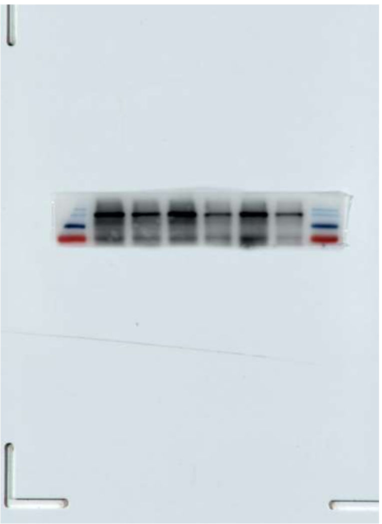

Fig6C β-actin

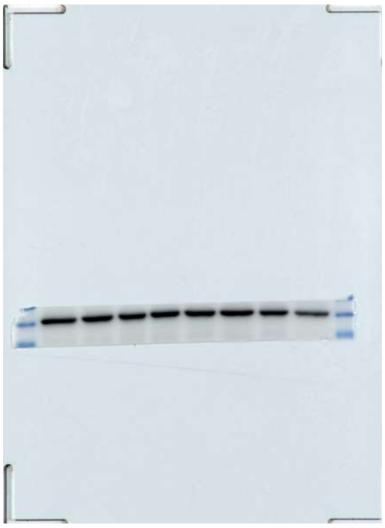

Fig6C E-cadherin

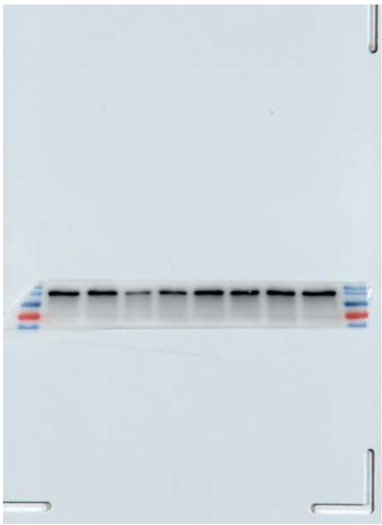

Fig6C SP-C

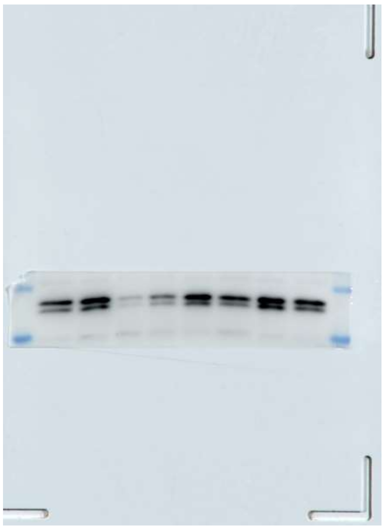

Supplement: Supplementary file 3 — Original Data File [file 41420_2024_1861_MOESM3_ESM.pdf]
